# Di-μ-azido-bis­({*N*′-[1-(2-pyrid­yl-κ*N*)ethyl­idene]acetohydrazidato-κ^2^ *N*′,*O*}dicopper(II))

**DOI:** 10.1107/S1600536810034860

**Published:** 2010-09-18

**Authors:** Amitabha Datta, Kuheli Das, Yan-Ming Jhou, Jui-Hsien Huang, Hon Man Lee

**Affiliations:** a National Changhua University of Education, Department of Chemistry, Changhua, Taiwan 50058

## Abstract

The dimeric title compound, [Cu_2_(C_9_H_10_N_3_O)_2_(N_3_)_2_], is located on a crystallographic inversion center. The Cu atom is coordinated by a tridentate anionic hydrazone ligand and two bridging azide ligands in a distorted square-pyramidal coordination geometry. The non-bonding Cu⋯Cu distance is 3.238 (1) Å. Non-classical inter­molecular C—H⋯N hydrogen bonds link the dimers into chains along the *c* axis.

## Related literature

For related dimeric copper(II) complexes with similar tridentate ligands, see: Recio Despaigne *et al.* (2009 ▶); Sen *et al.* (2007 ▶); Patole *et al.* (2003 ▶).
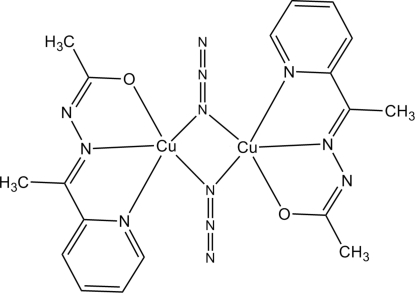


## Experimental

### 

#### Crystal data

[Cu_2_(C_9_H_10_N_3_O)_2_(N_3_)_2_]
*M* *_r_* = 563.54
Triclinic, 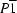

*a* = 7.589 (3) Å
*b* = 8.955 (3) Å
*c* = 9.693 (4) Å
α = 66.534 (15)°
β = 69.461 (13)°
γ = 81.468 (16)°
*V* = 565.8 (4) Å^3^
*Z* = 1
Mo *K*α radiation
μ = 1.92 mm^−1^
*T* = 150 K
0.25 × 0.20 × 0.20 mm

#### Data collection

Bruker SMART APEXII diffractometer
Absorption correction: multi-scan (*SADABS*; Sheldrick, 2003 ▶) *T*_min_ = 0.645, *T*_max_ = 0.700
3858 measured reflections
2358 independent reflections
1591 reflections with *I* > 2σ
*R*_int_ = 0.040

#### Refinement

*R*[*F* ^2^ > 2σ(*F* ^2^)] = 0.027
*wR*(*F* ^2^) = 0.093
*S* = 1.08
2358 reflections
156 parameters
H-atom parameters constrained
Δρ_max_ = 2.08 e Å^−3^
Δρ_min_ = −2.81 e Å^−3^

### 

Data collection: *APEX2* (Bruker, 2007 ▶); cell refinement: *SAINT* (Bruker, 2007 ▶); data reduction: *SAINT*; program(s) used to solve structure: *SHELXS97* (Sheldrick, 2008 ▶); program(s) used to refine structure: *SHELXL97* (Sheldrick, 2008 ▶); molecular graphics: *SHELXTL* (Sheldrick, 2008 ▶); software used to prepare material for publication: *DIAMOND* (Brandenburg, 2006 ▶).

## Supplementary Material

Crystal structure: contains datablocks I, global. DOI: 10.1107/S1600536810034860/pv2324sup1.cif

Structure factors: contains datablocks I. DOI: 10.1107/S1600536810034860/pv2324Isup2.hkl

Additional supplementary materials:  crystallographic information; 3D view; checkCIF report

## Figures and Tables

**Table 1:** Hydrogen-bond geometry (Å, °)

*D*—H⋯*A*	*D*—H	H⋯*A*	*D*⋯*A*	*D*—H⋯*A*
C9—H9*A*⋯N3^i^	0.98	2.74	3.710 (4)	171

Symmetry code: (i) 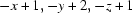.

**Fig. 1.**
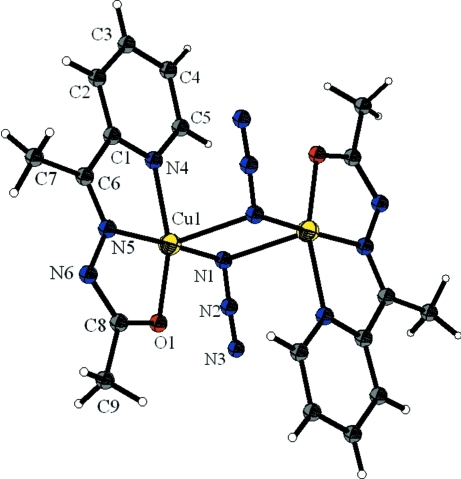
The structure of the title compound, showing 50% probability displacement ellipsoids for the non-hydrogen atoms. The unlabelled atoms are related to the labelled ones by symmetry operation: 1 - x, 2 - y, 2 - z.

**Fig. 2.**
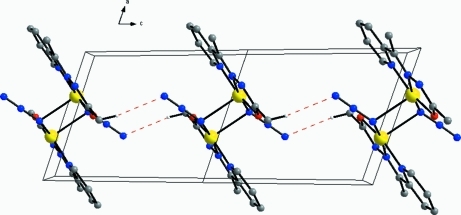
A view of the crystal packing along the b axis. Hydrogen bonds are shown as dashed lines and H-atoms not involved in H-bonds have been excluded for clarity.

## supplementary crystallographic information

### Comment

The title compound is a dimeric copper(II) complex. Each copper atom is coordinated by a tridentate, anionic hydrazone ligand and two bridging azide ligands. The non-bonding Cu···Cu distance is 3.238 (1) Å, which is slightly longer than that in a related dicopper azido complex (Sen *et al.*., 2007).

The dimer is located on a crystallographic inversion center. The non-classical intermolecular hydrogen bonds of the type C—H···N link the dimeric compounds into one dimensional chains along the *c* axis.

Dimeric copper(II) complexes with similar tridentate ligands have been reported in the literature (Recio Despaigne *et al.*, 2009; Sen *et al.* 2007; Patole *et al.* 2003).

### Experimental

The tridentate ligand precursor, 2-benzoylpyridine-methyl hydrazone, was prepared according to the literature procedure (Recio Despaigne *et al.*, 2009). To the tridentate ligand precursor (1.0 mmol), methanolic solution (20 ml) of copper nitrate trihydrate (0.241 g, 1.0 mmol), was added, followed by the addition, with constant stirring of a solution of sodium azide (0.065 g, 1.0 mmol) in minimum volume of water/methanol mixture. The final solution was kept at room temperature yielding brown square-shaped crystals suitable for X-ray diffraction after few days. Crystals were isolated by filtration and were air-dried.

### Refinement

All the hydrogen atoms were located in the difference Fourier map, nevertheless, all the H atoms were positioned geometrically and refined as riding atoms, with C_aryl_—H = 0.95, C_methyl_—H = 0.98 Å while *U*_iso_(H) = 1.2*U*_eq_(C_aryl_) and 1.5*U*_eq_(C_methyl_) H atoms. Although the residual electron in the final difference map is high, the refinement model appears to be reliable since the largest peak and hole are located near the heavy Cu atom at distances of 0.70 and 0.03 Å, respectively.

### Crystal data

**Table:**

[Cu_2_(C_9_H_10_N_3_O)_2_(N_3_)_2_]	*Z* = 1
*M**_r_* = 563.54	*F*(000) = 286
Triclinic, *P*1	*D*_x_ = 1.654 Mg m^−^^3^
Hall symbol: -P 1	Mo *K*α radiation, λ = 0.71073 Å
*a* = 7.589 (3) Å	Cell parameters from 3185 reflections
*b* = 8.955 (3) Å	θ = 2.9–28.5°
*c* = 9.693 (4) Å	µ = 1.92 mm^−^^1^
α = 66.534 (15)°	*T* = 150 K
β = 69.461 (13)°	Prism, brown
γ = 81.468 (16)°	0.25 × 0.20 × 0.20 mm
*V* = 565.8 (4) Å^3^	

### Data collection

**Table:**

Bruker SMART APEXII diffractometer	2358 independent reflections
Radiation source: fine-focus sealed tube	1591 reflections with *I* > 2σ
graphite	*R*_int_ = 0.040
ω scans	θ_max_ = 27.0°, θ_min_ = 3.1°
Absorption correction: multi-scan (*SADABS*; Sheldrick, 2003)	*h* = −9→9
*T*_min_ = 0.645, *T*_max_ = 0.700	*k* = −11→10
3858 measured reflections	*l* = −12→12

### Refinement

**Table:**

Refinement on *F*^2^	Primary atom site location: structure-invariant direct methods
Least-squares matrix: full	Secondary atom site location: difference Fourier map
*R*[*F*^2^ > 2σ(*F*^2^)] = 0.027	Hydrogen site location: difference Fourier map
*wR*(*F*^2^) = 0.093	H-atom parameters constrained
*S* = 1.08	*w* = 1/[σ^2^(*F*_o_^2^) + (0.0597*P*)^2^] where *P* = (*F*_o_^2^ + 2*F*_c_^2^)/3
2358 reflections	(Δ/σ)_max_ = 0.001
156 parameters	Δρ_max_ = 2.08 e Å^−^^3^
0 restraints	Δρ_min_ = −2.81 e Å^−^^3^

### Special details

**Table:**

Geometry. All e.s.d.'s (except the e.s.d. in the dihedral angle between two l.s. planes) are estimated using the full covariance matrix. The cell e.s.d.'s are taken into account individually in the estimation of e.s.d.'s in distances, angles and torsion angles; correlations between e.s.d.'s in cell parameters are only used when they are defined by crystal symmetry. An approximate (isotropic) treatment of cell e.s.d.'s is used for estimating e.s.d.'s involving l.s. planes.
Refinement. Refinement of *F*^2^ against ALL reflections. The weighted *R*-factor *wR* and goodness of fit *S* are based on *F*^2^, conventional *R*-factors *R* are based on *F*, with *F* set to zero for negative *F*^2^. The threshold expression of *F*^2^ > σ(*F*^2^) is used only for calculating *R*-factors(gt) *etc*. and is not relevant to the choice of reflections for refinement. *R*-factors based on *F*^2^ are statistically about twice as large as those based on *F*, and *R*- factors based on ALL data will be even larger.

### Fractional atomic coordinates and isotropic or equivalent isotropic displacement parameters (Å^2^)

**Table:**

	*x*	*y*	*z*	*U*_iso_*/*U*_eq_	
Cu1	0.34274 (3)	0.93665 (3)	0.95880 (3)	0.02989 (14)	
N5	0.2011 (2)	0.7390 (2)	1.0402 (2)	0.0307 (5)	
O1	0.4938 (2)	0.8357 (2)	0.8052 (2)	0.0380 (5)	
N1	0.4648 (3)	1.1498 (2)	0.8642 (2)	0.0329 (5)	
N6	0.2719 (3)	0.6294 (3)	0.9663 (2)	0.0367 (6)	
C8	0.4270 (3)	0.6937 (3)	0.8443 (3)	0.0314 (6)	
N2	0.5605 (3)	1.2073 (2)	0.7250 (2)	0.0358 (6)	
N4	0.1206 (2)	0.9791 (2)	1.1350 (2)	0.0315 (5)	
C1	−0.0012 (3)	0.8504 (3)	1.2187 (3)	0.0341 (6)	
C2	−0.1553 (3)	0.8504 (4)	1.3488 (3)	0.0438 (8)	
H2	−0.2404	0.7619	1.4036	0.053*	
C9	0.5264 (3)	0.5944 (3)	0.7465 (3)	0.0436 (7)	
H9A	0.4938	0.6357	0.6483	0.065*	
H9B	0.4881	0.4806	0.8067	0.065*	
H9C	0.6627	0.6019	0.7202	0.065*	
C5	0.0911 (3)	1.1048 (3)	1.1831 (3)	0.0390 (7)	
H5	0.1758	1.1935	1.1254	0.047*	
C6	0.0467 (3)	0.7153 (3)	1.1593 (3)	0.0351 (7)	
C3	−0.1850 (3)	0.9815 (4)	1.3992 (3)	0.0470 (9)	
H3	−0.2888	0.9825	1.4891	0.056*	
C4	−0.0596 (4)	1.1095 (4)	1.3148 (3)	0.0449 (7)	
H4	−0.0761	1.1997	1.3465	0.054*	
N3	0.6520 (4)	1.2683 (4)	0.5959 (3)	0.0644 (10)	
C7	−0.0748 (4)	0.5681 (4)	1.2321 (4)	0.0564 (10)	
H7A	−0.0411	0.5099	1.1594	0.085*	
H7B	−0.2073	0.6022	1.2516	0.085*	
H7C	−0.0556	0.4962	1.3328	0.085*	

### Atomic displacement parameters (Å^2^)

**Table:**

	*U*^11^	*U*^22^	*U*^33^	*U*^12^	*U*^13^	*U*^23^
Cu1	0.03078 (16)	0.0297 (2)	0.02843 (18)	−0.00779 (10)	−0.00486 (12)	−0.01154 (14)
N5	0.0319 (7)	0.0317 (10)	0.0289 (9)	−0.0042 (7)	−0.0062 (7)	−0.0133 (8)
O1	0.0400 (8)	0.0382 (10)	0.0350 (9)	−0.0100 (6)	−0.0044 (7)	−0.0163 (8)
N1	0.0386 (8)	0.0306 (10)	0.0287 (9)	−0.0063 (7)	−0.0068 (8)	−0.0117 (9)
N6	0.0383 (9)	0.0368 (11)	0.0351 (10)	−0.0048 (8)	−0.0079 (9)	−0.0154 (9)
C8	0.0389 (9)	0.0296 (11)	0.0318 (10)	−0.0017 (8)	−0.0140 (9)	−0.0148 (9)
N2	0.0404 (9)	0.0327 (10)	0.0316 (10)	−0.0034 (7)	−0.0105 (8)	−0.0091 (9)
N4	0.0303 (7)	0.0331 (10)	0.0310 (9)	−0.0027 (6)	−0.0079 (7)	−0.0126 (8)
C1	0.0286 (8)	0.0403 (12)	0.0295 (11)	−0.0040 (7)	−0.0073 (8)	−0.0098 (10)
C2	0.0346 (10)	0.0535 (16)	0.0353 (12)	−0.0070 (9)	−0.0012 (9)	−0.0150 (13)
C9	0.0495 (12)	0.0421 (15)	0.0402 (13)	−0.0022 (10)	−0.0097 (11)	−0.0200 (12)
C5	0.0395 (10)	0.0399 (14)	0.0386 (12)	−0.0032 (9)	−0.0109 (10)	−0.0162 (12)
C6	0.0323 (9)	0.0352 (12)	0.0343 (11)	−0.0074 (8)	−0.0070 (9)	−0.0102 (10)
C3	0.0388 (10)	0.0623 (19)	0.0358 (13)	0.0030 (10)	−0.0028 (10)	−0.0234 (14)
C4	0.0484 (12)	0.0489 (16)	0.0437 (14)	0.0071 (10)	−0.0130 (11)	−0.0277 (12)
N3	0.0746 (16)	0.0654 (19)	0.0323 (12)	−0.0125 (13)	−0.0042 (12)	−0.0045 (14)
C7	0.0493 (13)	0.0495 (18)	0.0599 (19)	−0.0198 (12)	0.0053 (13)	−0.0229 (16)

### Geometric parameters (Å, °)

**Table:**

Cu1—N5	1.941 (2)	C1—C6	1.484 (4)
Cu1—N1	1.969 (2)	C2—C3	1.403 (5)
Cu1—O1	1.979 (2)	C2—H2	0.9500
Cu1—N4	2.051 (2)	C9—H9A	0.9800
Cu1—N1^i^	2.4574 (18)	C9—H9B	0.9800
N5—C6	1.297 (3)	C9—H9C	0.9800
N5—N6	1.377 (4)	C5—C4	1.394 (4)
O1—C8	1.300 (3)	C5—H5	0.9500
N1—N2	1.218 (3)	C6—C7	1.500 (3)
N1—Cu1^i^	2.4574 (18)	C3—C4	1.386 (4)
N6—C8	1.341 (3)	C3—H3	0.9500
C8—C9	1.493 (5)	C4—H4	0.9500
N2—N3	1.142 (3)	C7—H7A	0.9800
N4—C5	1.343 (4)	C7—H7B	0.9800
N4—C1	1.374 (3)	C7—H7C	0.9800
C1—C2	1.387 (4)		
			
N5—Cu1—N1	173.44 (6)	C1—C2—C3	119.8 (2)
N5—Cu1—O1	79.78 (9)	C1—C2—H2	120.1
N1—Cu1—O1	101.14 (9)	C3—C2—H2	120.1
N5—Cu1—N4	80.27 (9)	C8—C9—H9A	109.5
N1—Cu1—N4	98.12 (10)	C8—C9—H9B	109.5
O1—Cu1—N4	159.42 (8)	H9A—C9—H9B	109.5
N5—Cu1—N1^i^	99.67 (7)	C8—C9—H9C	109.5
N1—Cu1—N1^i^	86.64 (6)	H9A—C9—H9C	109.5
O1—Cu1—N1^i^	98.75 (8)	H9B—C9—H9C	109.5
N4—Cu1—N1^i^	89.51 (8)	N4—C5—C4	122.3 (2)
C6—N5—N6	123.0 (2)	N4—C5—H5	118.9
C6—N5—Cu1	119.7 (2)	C4—C5—H5	118.9
N6—N5—Cu1	117.28 (14)	N5—C6—C1	113.21 (19)
C8—O1—Cu1	110.27 (16)	N5—C6—C7	124.6 (3)
N2—N1—Cu1	122.4 (2)	C1—C6—C7	122.2 (3)
N2—N1—Cu1^i^	111.79 (13)	C4—C3—C2	118.5 (3)
Cu1—N1—Cu1^i^	93.36 (6)	C4—C3—H3	120.7
C8—N6—N5	107.9 (2)	C2—C3—H3	120.7
O1—C8—N6	124.6 (3)	C3—C4—C5	119.4 (3)
O1—C8—C9	118.6 (2)	C3—C4—H4	120.3
N6—C8—C9	116.8 (2)	C5—C4—H4	120.3
N3—N2—N1	176.3 (3)	C6—C7—H7A	109.5
C5—N4—C1	119.0 (2)	C6—C7—H7B	109.5
C5—N4—Cu1	128.98 (15)	H7A—C7—H7B	109.5
C1—N4—Cu1	111.8 (2)	C6—C7—H7C	109.5
N4—C1—C2	121.1 (3)	H7A—C7—H7C	109.5
N4—C1—C6	114.9 (2)	H7B—C7—H7C	109.5
C2—C1—C6	124.0 (2)		

Symmetry codes: (i) −*x*+1, −*y*+2, −*z*+2.

### Hydrogen-bond geometry (Å, °)

**Table:**

*D*—H···*A*	*D*—H	H···*A*	*D*···*A*	*D*—H···*A*
C9—H9A···N3^ii^	0.98	2.74	3.710 (4)	171

Symmetry codes: (ii) −*x*+1, −*y*+2, −*z*+1.
